# Barriers and Innovations Towards Accessing an Autism Diagnosis in Rural Northern Ontario: A Qualitative Study

**DOI:** 10.1111/cch.70250

**Published:** 2026-03-05

**Authors:** Carly A. Cermak, Jesiqua Rapley, Sherry Fournier, Melanie Penner

**Affiliations:** ^1^ Bloorview Research Institute Holland Bloorview Kids Rehabilitation Hospital Toronto Ontario Canada; ^2^ Child & Community Resources Sudbury Ontario Canada; ^3^ Division of Developmental Pediatrics, Department of Pediatrics University of Toronto Toronto Ontario Canada

**Keywords:** child development, culturally sensitive care, early identification, inter‐professional collaboration

## Abstract

**Background:**

Early identification and diagnosis of autism are essential steps in supporting children and families. In rural communities, families can experience significant challenges in accessing an autism diagnosis.

**Methods:**

We conducted semistructured interviews with four groups of participants: managers (*n* = 11), diagnosticians (*n* = 7), clinicians (*n* = 14) and parents/caregivers (*n* = 20) across six regions in Northern Ontario to learn of barriers and facilitators in accessing an autism diagnosis. Two independent coders coded each transcript and used inductive thematic analysis to identify themes across participants and regions.

**Results:**

Five themes were generated from participants: (1) Gaps in autism knowledge delay early identification for children requiring an autism assessment; (2) complex systems create navigation barriers for families in knowing where to seek help; (3) families with limited access to resources face delays in the early identification process; (4) staffing challenges exacerbate barriers within the autism diagnostic process; and (5) collaboration among health disciplines while using a culturally responsive approach to care facilitates the autism assessment process.

**Discussion:**

Hopes about the future of accessing an autism diagnosis were shared by families and professionals; although some challenges are fixed (e.g., vast geography), others are amenable to interventions such as building community knowledge and streamlining service navigation.

## Summary

- Community‐level knowledge of autism can support early identification of children requiring a developmental assessment.
- Referrals for an autism assessment can be delayed when parent concerns about their child's development are not taken seriously by physicians.
- Community clinics (i.e., where health care professionals go into rural communities) support families who may not have access to transportation or internet capabilities (e.g., Wi‐Fi access) for virtual options.
- Interprofessional collaboration and communication among health care professionals of varying disciplines facilitate the autism diagnostic process.
- Culturally responsive care supports families in understanding their values and beliefs while also facilitating appropriate next steps for the child.

## 1 Background

Autism spectrum disorder (ASD; autism) is a neurodevelopmental condition characterized by differences in social communication and evidence of restricted, repetitive behaviours (American Psychiatric Association 2022). Approximately one in 66 Canadians is autistic, with a significantly higher prevalence rate in males than females (Ofner et al. 2018). Early identification of neurodevelopmental differences is a critical first step in a child's care pathway (Zwaigenbaum et al. 2015), as intervention provided early in development can support a child's cognitive, behavioural and communication outcomes (Hampton and Kaiser 2016; Nahmias et al. 2019). In Ontario, a diagnosis of autism is required to access publicly funded programmes, with most intervention programs focused on the early years (i.e., 4 years of age or younger). Taken together, early identification of autism features is vital for a child to get a diagnosis and access the supports they require for a successful entry into school.

Parents, caregivers and early childhood professionals play a vital role in helping recognize developmental differences in children. However, geographical constraints can impact timely identification, particularly if there is a lack of autism awareness and knowledge (Antezana et al. 2017). Social determinants of health such as community needs and financial limitations also play a role in later identification (Antezana et al. 2017). Further, a lack of qualified health care providers in rural regions can impact parents' navigation of where to seek help (Vanegas et al. 2023). Taken together, rurality may have an impact on timely identification, particularly if there is a lack of knowledge about autism in the community, few healthcare professionals and social needs within families.

Access to autism diagnosis is challenging across Canada (Penner et al. 2018); however, the geography, population distribution and health care workforce challenges in rural areas further complicate this. In some cases, families endure sequential wait times; a primary care physician refers to a paediatrician who then refers to a subspecialist in order to obtain a diagnosis (Penner et al. 2017). Too often, subsequent referrals to specialty clinics prolong wait times, with families waiting years for an autism diagnosis (Penner et al. 2018). In rural regions, finding trained physicians and healthcare professionals who can make a diagnosis or make a referral to a subspecialty clinic is difficult (Antezana et al. 2017; Hoogsteen and Woodgate 2013; Murphy and Ruble 2012). Consequently, children living in rural communities are diagnosed at a later age than those in urban communities (Lauritsen et al. 2014).

Exploration of factors contributing to accessing an autism assessment in regions with a dispersed geographical landscape is warranted, as geographical barriers present a unique challenge that may require specific strategies related to improving timely access to diagnostic services. To date, there are few studies examining the diagnostic process in Canada. Das et al. (2022) interviewed paediatricians in three rural communities in Ontario; however, this study did not capture the perspectives of other interest holders in the diagnostic process (e.g., nonphysician clinicians and families). Young et al. (2019) conducted focus groups with community interest holders in British Columbia and Alberta to gain a better understanding of issues in autism services; however, their focus was on the postdiagnostic stage in autism care rather than the pre‐diagnostic stage. Thus, further exploration of factors impacting the diagnostic process in Canada is warranted, particularly in regions with a vast geographical landscape.

The objectives of our study were (1) to describe current approaches of accessing autism diagnosis in Northern Ontario, including innovative approaches and their impact, and (2) to understand ongoing access gaps, opportunities and required resources to optimize timely autism diagnostic access in Northern Ontario.

## 2 Methods

### 2.1 Setting

Northern Ontario makes up over 80% of Ontario's land (806 000 km^2^), yet only houses a tiny fraction of the province's population at approximately 800 000 people (1 person per km^2^). This contrasts with Southern Ontario, with a population of over 13 million people in an area of 112 000 km^2^ (Government of Canada 2022). The prevalence of autism in Ontario for children ages 1–17 years falls around 2%, consistent with the overall prevalence of autism in Canada (Public Health Agency of Canada 2022); rates specific to Northern Ontario are not available. Notably, Northern Ontario is comprised of many First‐Nations communities who require fly‐in access, adding an important cultural element to the autism diagnostic process (Antony et al. 2022; Bruno et al. 2024, 2025).

### 2.2 Community Partnership

An advisory group with members from different regions in Northern Ontario was formed to connect and discuss the current state of paediatric disability care, particularly access to autism diagnosis. Advisory group members included managers, diagnosticians and clinicians who work in child development services in Northern Ontario, as well as two parent advocates who have been through the autism diagnostic process in Northern Ontario. The Northern Ontario Advisory Group reached out to this research team, based in Toronto (Southern Ontario), to discuss and support the development of the study design, the recruitment strategy and the interview guide. The research team consisted of a female speech–language pathologist and research associate (C.A.C.), a female research assistant (J.R.), a female executive director of child and community services (S.F.) and a female developmental paediatrician and autism researcher (M.P.). The reporting of this study was guided by the Standards for Reporting Qualitative Research (O'Brien et al. 2014).

### 2.3 Recruitment

This work was approved by the Research Ethics Board of the Bloorview Research Institute. Purposive sampling was used to recruit four groups of participants. Recruitment started with members of the advisory group sharing the study flier broadly through their networks for all study groups. Leadership professionals (Group 1) disseminated study information to their respective diagnosticians (Group 2) and community clinicians (Group 3). From there, community clinicians assisted in the recruitment of parents/caregivers (Group 4) by informing them through their networks, along with an invitation to contact the research team if they were interested in participating either through a flyer or a recruitment email. Parents/caregivers were eligible to participate if they had gone through the autism diagnostic process within the last 5 years; a diagnosis of autism was not an inclusion criterion for this study.

### 2.4 Data Collection

All participants gave written, informed consent prior to the interview. Participants were asked to fill out a brief demographic survey to capture characteristics of the sample and facilitate purposive sampling to ensure demographic representation across groups, particularly families. This was followed by a 45‐min semistructured interview via Zoom Healthcare. Interview guides were specifically developed for each group (e.g., managers, diagnosticians, clinicians and families). The interview guide for managers was developed to understand current processes (e.g., ‘What does your diagnostic process look like?’ and ‘How is your team doing with the current process?’). The interview guides for the groups of diagnosticians and clinicians were developed using the COM‐B, a framework designed to examine why people do or do not do certain behaviours (B) based on their capabilities (C), opportunities or environment (O) and their motivation (M) to change (Michie et al. 2011). Examples of interview questions from each component of the COM‐B included (1) capability (e.g., ‘What knowledge do you have about autism?’), (2) motivation (e.g., ‘Do you feel confident in your skills to identify children with possible autism?’) and (3) opportunity (e.g., ‘What factors are impacting the current process in supporting autism identification and diagnosis?’). The interview guide for families was developed to understand parent/caregiver experiences in three phases of the diagnostic process: (1) preassessment (e.g., ‘What was your knowledge of how to get an autism assessment?’ and ‘Why did you seek help when you did and what did that look like?’), (2) during the assessment (e.g., ‘What did the assessment look like?’) and (3) postassessment (e.g., ‘What information were you given regarding next steps?’ and ‘How confident were you in knowing what to do next?’). Parents/caregivers were also asked a question about future hopes, including ‘If you were going through this process again, what would you have hoped been done differently?’ and ‘What change would you like to see in the process for accessing autism diagnosis moving forward?’ (see Supporting Information S1).

All interviews were completed by the first author (C.A.C.) between February and April 2024. Interviews were audio recorded and transcribed using Zoom transcription. Data collection was concluded when saturation of themes was reached, meaning that limited new insights emerged from existing themes with the collected data sample (Saunders et al. 2018).

### 2.5 Data Analysis

The qualitative, narrative research design was conceived within an interpretivist paradigm, where the researchers' purpose was to gather insight into how the autism diagnostic process is working in Northern Ontario (Thanh and Thanh 2015). Interviews were analysed using an inductive thematic analysis approach, which included openly coding line by line to organize data in a meaningful, systematic way (e.g., preassessment, assessment and postassessment); examining the codes to identify themes; and reviewing the themes (Braun and Clark 2022; Braun and Clarke 2006). Specifically, two researchers independently coded each transcript and met twice per week to review codes, discuss themes and develop a code book. From here, the researchers continued to meet on a weekly basis to ensure the reliability of coding. Reflexivity occurred through the weekly meetings to review the codes and modify the codebook (i.e., adding new codes) as the groups of participants expanded (e.g., from managers to diagnosticians to clinicians to families). Further, initial themes were presented to the advisory group for feedback. NVivo software was used to manage data and facilitate the cross‐synthesis of data.

## 3 Results

A total of 52 participants (11 managers, 7 diagnosticians, 14 clinicians and 20 parents/caregivers) were interviewed across six regions in Northern Ontario: Algoma, Cochrane (including Temiskaming), Kenora (including Rainy River), Nipissing, Sudbury (including Manitoulin) and Thunder Bay. Diagnosticians included general paediatricians (*n* = 3), psychologists (*n* = 2), psychologist associates (*n* = 1) and developmental paediatricians (*n* = 1). Clinicians included Infant Development or Family Preservation Worker (*n* = 6), Occupational Therapy (*n* = 4), Social Work (*n* = 2), Behaviour Therapy (*n* = 1) and Speech–Language Pathology (*n* = 1). To avoid identification, the groups of managers, diagnosticians and clinicians are hereafter referred to as ‘professionals’, and the group of parents/caregivers is hereafter referred to as ‘parents’ or ‘families’ (see Table 1).

**TABLE 1:** Demographics of study participants (*N* = 52).

	Professionals (*n* = 32)	Parents/caregivers (*n* = 20)
Gender		
Woman	28	20
Man	4	0
Age (mean in years, SD)	44.7 (9.9)^a^	37.5 (5.0)^b^
Race/ethnicity		
Indigenous	1	4
Latin‐American/Hispanic	0	1
Mixed	2	0
White/European origin	29	15
District		
Algoma	7	5
Cochrane/Temiskaming	3	3
Kenora/Rainy River	5	2
Nipissing/Parry Sound/Muskoka	6	7
Sudbury	2	0
Thunder Bay	9	3

Abbreviation: SD: standard deviation.

^a^ *n* = 11 did not provide age.

^b^ *n* = 5 did not provide age.

Results from participant interviews revealed five themes that were present across districts: (1) Gaps in autism knowledge delay early identification of children requiring an autism assessment; (2) complex systems create navigation barriers for families in knowing where to seek help; (3) families with limited access to resources face delays in the early identification process; (4) staffing challenges exacerbate barriers within the autism diagnostic process; and (5) collaboration among health disciplines, while using a culturally responsive approach to care, facilitates the autism assessment process.

Although these themes were consistent across districts in Northern Ontario, many barriers identified within the autism diagnostic process (e.g., knowledge gaps, staffing, technology and resources) were exacerbated in remote, fly‐in communities and rural communities (i.e., 3‐h drive to the closest city/hub). The element of rurality adds to the complexities of access across all levels of service from daycares to doctors, highlighting the unique needs of populations that reside in remote regions.

1 Gaps in autism knowledge delay early identification of children requiring an autism assessment.

Knowledge gaps described by professionals and families included decreased awareness of autism in Northern Ontario communities. Gaps in knowledge were felt by all participant groups to impact timely identification. For professionals, they described inconsistencies among educators and health care workers in recognizing autism features.

There's a variability, and the amount of comfort nurses have with recognizing red flags of autism. But I think that's true even among general practitioners, right? So, yeah, it's extremely variable. (ID 21, Professional)

For families, they described feeling frustrated that their concerns were not validated by their physician. In other words, if their child did not demonstrate evident features of autism in the physician's office, a referral for an autism assessment was not made or the physician adhered to a ‘wait‐and‐see’ approach.

… because we are so isolated from other areas, there's a lot of misinformation … because there's the kind of stereotype autism versus the spectrum of what it could be … and that leads to a lot more later diagnosis. (ID 11, Parent)

Professionals described the need for more service providers to host education sessions about autism within the community, whereas families suggested putting pamphlets and posters about autism in doctor's offices and community centres.

2 Complex systems create navigation barriers for families in knowing where to seek help.

I do not think parents are aware about the signs … I think there needs to be more awareness of autism itself in general to the public and to parents. And I do not know if that starts at the doctor's … at public health … But just because parents do not have the information to even make the decisions, they just do not know. (ID 13, Parent)

Families and professionals described the complexities in knowing where to go to seek help. In some districts, professionals described improvements that have been made to identification and referral processes such as the use of developmental screening tools, health care professionals dropping into community centres to make connections with parents and caregivers and agencies implementing developmental monitoring programs for siblings of children who have received a diagnosis. Despite these improvements, many professionals continue to describe the complexities of service navigation for families.

… it's extremely confusing as a parent to understand where you have to go, when you have to go, why you have to go, and we often overlook that the families up here do not have capacity and education and the ability to sometimes understand these really complex service systems. (ID 8, Professional)

… I just feel so bad for these families because I'm like you have just gone through … this life‐changing diagnosis, and it's difficult. And you are just trying to live day to day. And then you also have to navigate through this confusing system that even the clinicians that are in that system have a hard time keeping. (ID 51, Professional)

Professionals described trying to bridge gaps between diagnosis and support due to the paucity of services available for children postdiagnosis. One staff member described

3 Families with limited access to resources face delays in the early identification process.

… I think there's a general you know, acceptance that wait lists are what they are for assessment. But it's the, you know… there's just so many gaps … I feel pressured to try to fill those gaps … And I'm always kind of stretching the limits of that you know, to try to offer that additional help and support. And yeah, we feel like, we are just not really making a difference. We have so few options for those tangible physical interventions for families. And it's just that the community is at such a loss for what to do especially once there's a diagnosis. (ID 1, Professional)

Professionals described the disparities between rural and urban communities, with rural communities having less access to daycares and family doctors who can help with early identification. Consequently, this results in health care professionals trying to navigate assessment and supports for children at a later age.

… we are often not getting these kids referred to us until four, five, six, because they do not have family doctors. They do not have other services involved in their care. They have not been seen in a daycare setting so we are getting referred these kids later, which means we have a lot more navigating to do at four, five, and six. (ID 30, Professional)

Participants across groups described the vast geographical landscape in Northern Ontario and the length of travel time (e.g., 3 h) to get to appointments. Professionals described that most of travel occurs in the late spring, summer and fall, as roads are closed during the winter months. For remote regions in the Far North, travel to community clinics occurs by plane, which can pose extra challenges for children with higher support needs. Further, not all families have a mode of transportation to get to diagnostic clinics, exacerbating challenges in accessing an assessment.

… our district size. It's also a challenge … not every family has the means or a mode of transportation to get here, so that could be really difficult. (ID 9, Professional)

… the biggest challenge there [in the far North] is travel because some of the kids do not like traveling. Some of them have a real phobia of flying in planes, and the parents often have to get some kind of sedation to give the child to bring them down. (ID 12, Professional)

Limitations to technology such as Wi‐Fi bandwidth and cellular service contribute to challenges in service access (e.g., virtual care) as well as informing families of appointments. Additionally, financial challenges may intensify family stressors, with these combined social determinants of health described by professionals as contributors to missed appointments and delays in the assessment process.

The biggest challenge we face … is communication, because some of them [the parents] do not have telephones. The only way you can communicate with them is through like a Wi‐Fi system … So we end up having to do Zoom calls through the school or through the clinic nursing station. Right? So it can be really challenging sometimes to do this. (ID 12, Professional)

Families also described having to home school their children due to the lack of appropriate supports in schools. This creates financial stress within families, with many mothers having to quit their jobs to be fulltime care takers, educators and therapists for their children.

4 Staffing challenges exacerbate barriers within the autism diagnostic process.

I now home school all three of my kids, because we cannot get them the proper help in the school system and with the new programme, I cannot afford for them to each have a therapist in the school. So yeah, it was a lot harder for her to get her diagnosis, and she has not had one drop of therapy even though she's diagnosed because…she's still waiting on funding. (ID 37, Parent)

Despite efforts to improve preassessment processes, a lack of diagnosticians (e.g., paediatricians and psychologists) and health care professionals more broadly remains a significant challenge in Northern Ontario. Consequently, staffing challenges slow down the pace at which children are seen, with professionals describing the need to hire external diagnosticians to travel in and help support the autism assessment process.

So in our area in particular, we have [a] really hard time with recruitment and retention of staff. … we get a lot of clinicians that come and just get, you know their experience. Get a year or two under their belt, and then they go back to where they are from usually like Winnipeg or sometimes Southern Ontario. So that's been a big challenge. We have been unable to fill vacancies…So we are a skeleton crew up here. So we just kind of do what we can. And yeah, unfortunately, that means that the kids are waiting long periods of time. (ID 43, Professional)

Further, staffing shortages mean that current staff are being stretched within their role, with many early intervention workers (e.g., Infant Development services and Family Preservation services) going beyond their role to support families (e.g., transportation, literacy and attending appointments).

… we wear lots of different hats as child development workers like you said…We're driving. We're going to doctors' appointments. We're doing ABA [applied behaviour analysis]. We're bringing them to [early child development] centers. We're in the schools. We're their everybody until somebody else can step in and help us. But … we are running short. (ID 28, Professional)

In addition to staffing challenges, professionals expressed their frustrations with assessment constraints, as provincial funding models impact what school board psychologists can assess and diagnose (e.g., learning needs vs. developmental differences). The leads to intricate and complicated situations, such as a child being on two different waitlists for two different assessments to see the same diagnostician that is hired by two different agencies.

… we have kids [for whom] we'll assess for this, and then we'll move them to this next list, and we'll assess for this and move them to this next list, and we are ending it up with a lot of kids around here who have … multiple diagnostic [assessments] … They're flip flopping through their diagnoses. And they end up in these really complex crisis situations. (ID 8, Professional)

Hopes expressed by professionals included expanding diagnostic roles, such as school psychologists being able to diagnose autism, and for more physicians and nurse practitioners (NPs) to gain clinical confidence to diagnose autism. These hopes were heard across regions as Northern Ontario is faced with a limited number of paediatricians and psychologists who diagnose autism.

5 Collaboration among health disciplines, while using a culturally responsive approach to care, facilitates the autism assessment process.

Interprofessional collaboration was paramount in supporting the diagnostic process, particularly when information from service providers was provided to diagnosticians to support clinical decision making. This was especially important in decision‐making for clinically complex children.

There's, you know, we talk about mercury poisoning in some of our First Nations. Intergenerational trauma. There's a lot of … co‐morbidities‐ it's not as easy as I think it might be in some other places like maybe central Southern Ontario, to go to a physician and get a diagnosis of ASD. The kids here often are … much more complex … (ID 2, Professional)

… when there is pediatrician involved, we are connecting with them. And there have been times where we have done observations and provided feedback to them, and then they have been able to go ahead from their perspective and make a diagnosis or not. Just based on the support that we have been able to give them. (ID 3, Professional)

Within the assessment process, culturally responsive care was at the forefront, with professionals describing the importance of meeting families where they are at.

… something I really value is that collaboration with the service partners … even the schools…So even being able to reach out to the schools and say, ‘Hey, you referred this kiddo?’…We've had schools really be supportive, like, ‘Let's do the assessment at the school, because that will work best for the family, for the child … We've got people here that can help them like, just be with them through the assessment.’ So that's been, I think one of the big wins for us is just that working together. (ID 48, Professional)

Further, professionals reported how they incorporated culturally responsive care within the Indigenous culture. This included modifying traditional standardized assessments such as the Autism Diagnostic Observation Schedule (ADOS) and relying on clinical observations of the child's social communication and behaviour to enhance the ecological validity of their approach.

It's just a very different worldview perspective when … within the Indigenous belief there's … no concept of disability, it's not something that can exist. And every child is brought by the Creator for a purpose, and chooses their family, and is meant to be, and will develop how they are meant and supposed to be and into their fullest potential. And so when you look at the process through that lens, often the need is really pragmatic … And so I try to take an [assessment] approach that honors and respects that… (ID 52, Professional)

Professionals working within these communities described the trust in health care providers that has been built over time with more families within First Nations communities accepting of the diagnostic process.

… [families are] more accepting of us and recognizing that we are not here just to label your child. We're here to try and help and improve the prognosis potentially, and improve the understanding of others, not just to change your child, but to change the acceptance of your child in the community and in the schools. (ID 12, Professional)

Families expressed their appreciation for health care providers coming into their community, as it reduces transportation barriers and lets health care providers see their child in a more comfortable setting. One diagnostician reported

… I feel it's so important for me to go there, because flying me into a [rural] community to see all … the kids there rather, than flying the kids to a [community site] … makes a lot more sense. Even though it is more challenging to work in those rural communities, because you do not have the same resources. (ID 21, Professional)

Postdiagnosis, families expressed how helpful support was for service navigation and funding applications; it reduced overwhelm and provided families with emotional support. However, all participants expressed the gaps in services postdiagnosis and the need for extended mental health supports for both child and family. Families also expressed hopes for educators to have basic autism training (e.g., how to communicate and address interfering behaviour) as this could reduce the stress and workload of educational assistants who are currently assigned to support autistic children.

Professionals identified contributors to service gaps including staffing challenges, technology limitations to offer virtual services and travel restrictions. One professional suggested implementing social skills interventions within the classroom as this could reduce the need to find service providers for social skills programs in the community.

## 4 Discussion

This qualitative study provides insight into the triumphs and challenges that Northern Ontario faces related to autism diagnosis, and the hopes expressed by professionals and parents on ways to improve it. Canadian literature has focused on perspectives of rural paediatricians on diagnosing autism (Das et al. 2022) and community perspectives on autism services (Young et al. 2019); however, our study provides cross‐cutting perspectives of professionals and families about accessing an autism diagnosis in a vast region within Ontario. We identified knowledge gaps, social determinants of health and system navigation as barriers to accessing an autism diagnosis. These barriers were exacerbated by Northern Ontario's sparse geography, technology limitations and lack of health care providers. An additional barrier included constraints within assessment scope (e.g., assessing learning needs only without also looking at developmental differences), a challenge experienced across Ontario due to funding models within the provincial government (Ministry of Children, Community and Social Services: Spending Plan Review 2024). Despite these barriers, there were evident facilitators to the diagnostic process including inter‐professional collaboration, culturally sensitive care, community visits and support with service navigation.

Knowledge gaps were a recurrent theme within our study findings and a contributing factor to later identification. In order to identify children who require further assessment, public and professional knowledge about autism and its associated features is essential. Knowledge gaps in autism continue to exist across North America (Corden et al. 2022; Golson et al. 2022; McCormack et al. 2020), despite a rising increase in incidence (Ofner et al. 2018). Suggestions made by parents to improve public awareness of autism included pamphlets and posters in community spaces and universal developmental screening. Although universal screening with an autism‐specific lens has been found to be effective for surveillance of autism features in community health and early education settings (Mozolic‐Staunton et al. 2020), the use of a highly sensitive autism‐specific screening tool (with comparatively lower specificity) can lead to a large volume of children with a false positive referral (Yuen et al. 2018). The Canadian Paediatric Guidelines recommend the use of autism‐specific screening only when parents, caregivers and/or professionals working with the child have identified concerns about the child's development through developmental surveillance (Williams et al. 2011; Zwaigenbaum et al. 2019). However, many parents in our study described feeling dismissed by physicians after expressing concerns about their child. Beyond the use of a screening tool, our results show the tensions that can exist between physicians and parents when discussing the possibility of autism. Physicians should engage in shared decision making with families in these situations, being mindful of wait times and age‐based limitations on accessing certain programs.

Parents feeling dismissed by physicians about their concerns with their child's development is a common occurrence across North America (Camden et al. 2020; Elder et al. 2016; Wong et al. 2017). Parents describe community physicians not understanding autism and what to look for, with many adhering to a wait and see approach (Elder et al. 2016; Wong et al. 2017). For parents who are unsure how to interpret their observations of their child, they rely on the expertise of the physician to determine if their observations warranted concern (Camden et al. 2020). Unfortunately, many community physicians continue to lack the knowledge of autism features needed to make appropriate referrals, make a diagnosis and/or validate parent concerns (McCormack et al. 2020; Wong et al. 2017). For rural communities that have few paediatricians, like Northern Ontario, it is imperative to utilize primary care physicians and NPs as diagnosticians (Hine et al. 2019). Research has demonstrated that clinicians can correctly identify autism in young children with a high level of accuracy (McNally Keehn et al. 2023; Penner et al. 2023; Wieckowski et al. 2021). Ruling out autism is harder, suggesting that more clinically complex children may be a better fit for a specialty clinic or diagnostic centre and/or with a multi‐disciplinary team (Penner et al. 2023; Wieckowski et al. 2021). Taken together, significant efforts are warranted to build capacity in autism assessment for community physicians and NPs. Collegial, interactive learning, such as seen in an Extension for Community Healthcare Outcomes Autism Program, or ECHO Autism (Becevic et al. 2021; Mazurek et al. 2017), can build the confidence physicians and NPs need to diagnose children with more evident features of autism and refer on to a speciality clinic for children with less evident features of autism.

Collaboration is an essential element to the autism diagnostic process (Brian et al. 2019) and may support clinical decision making for complex cases. The importance of collaboration was paramount in our study findings, with inter‐disciplinary communication at the forefront of facilitating the diagnostic process. Consistent with Das et al. (2022), information from community providers (e.g., educators) and allied health disciplines (e.g., Speech–Language Pathology and Occupational Therapy) provided diagnosticians with clinically relevant background information about the child and family to support clinical decision making. Collaboration with communities and families was also foundational for building relationships with remote, fly‐in First Nations communities. Diagnosticians and clinicians described their approach to autism assessment with First Nations communities, such as using an ecological approach to assessment (e.g., observations in community settings) and learning from families about what is important to them and their culture. This approach supports the growing recognition for trauma‐informed, culturally sensitive care that incorporates an understanding of the history context of Indigenous communities (Antony et al. 2022). Further, our findings illustrate that meaningful interactions can be built over time when there are culturally sensitive approaches put in place between trusted community providers and families.

Navigation within the autism diagnostic process is complex and involves many steps including locating, coordinating and navigating care (Mazurek et al. 2024). Parents in our study described how helpful it was to have service navigation support postdiagnosis, expressing the need for a more streamlined approach to postdiagnostic services if service navigation support is not available. However, the lack of support postdiagnosis was evident, with many parents describing having to leave their job to take care of their children due to unsupportive school environments. Further, parents expressed the lack of mental health support for their child, with children having no one to turn to, to help their emotional state. Our findings are consistent with the experiences of families in rural regions in the United States and other regions of Canada including the need for a ‘one stop shop’, the financial hardships in leaving jobs to take care of autistic children and the lack of mental health support for autistic children (Vanegas et al. 2023; Young et al. 2019). What was unique to Northern Ontario was the technology barriers (e.g., Wi‐Fi) that exacerbated service barriers, with bandwidth restrictions limiting access to virtual care. Taken together, the challenges in navigating services and accessing resources contribute to the social isolation families living in rural regions experience (Ault et al. 2021). Greater attention needs to be paid to the overall wellbeing of caregivers with autistic children, particularly in rural regions where social networks may be few.

## 5 Limitations

Our study was exploratory with findings drawing on descriptive, qualitative data within communities in Northern Ontario, Canada. The generalizability of findings should be considered in relation to each community's unique characteristics, resources and funding models. Furthermore, there was not equal representation from each district or representation from fathers on their experiences; however, the Northern Ontario advisory group who helped guide this work felt that the identified themes were representative of the challenges they face in accessing an autism diagnosis.

## 6 Conclusion

Northern Ontario is faced with unique challenges given its vast geographical landscape, lack of diagnosticians and limitations to technology (e.g., Wi‐Fi). Factors that exacerbate these barriers include knowledge gaps, system and service navigation (i.e., parents knowing where to get help), social determinants of health and community needs. Professional and community collaborations remain at the forefront of facilitating the diagnostic process, demonstrating the power of partnership in autism assessments. However, continued capacity building in autism knowledge for diagnosticians and the general community remains a priority so children who need a developmental assessment can be identified and evaluated. Additionally, more streamlined access to postdiagnosis support is warranted to facilitate service navigation and mental health supports.

## Author Contributions

**Carly A. Cermak:** methodology, data curation, formal analysis, writing the original draft. **Jesiqua Rapley:** formal analysis, writing – review and editing. **Sherry Fournier:** conceptualization, resources, writing – review and editing. **Melanie Penner:** conceptualization, supervision, project administration, writing – review and editing.

## Funding

This study received financial support from Empowered Kids Ontario.

## Ethics Statement

The Research Ethics Board of the Bloorview Research Institute approved this study (REB # 0648).

## Consent

Informed consent to participate was written and verbal.

## Conflicts of Interest

The author (M.P.) declares current grant funding from CIHR and previous funding from Autism Speaks. Additionally, previous paid consulting with Addis & Associates/Roche and for the Province of Nova Scotia.

## Supporting information


**Data S1:** Supporting information.

## Data Availability

Transcript data cannot be shared due to participant privacy and confidentiality. The study codebook is available upon request.
