# Supplementary material for: Barriers and Innovations Towards Accessing an Autism Diagnosis in Rural Northern Ontario: A Qualitative Study
Source: Child Care Health Dev. 2026 Mar 5;52(2):e70250. doi: 10.1111/cch.70250 (PMC12962844; doi:10.1111/cch.70250)
Supplement: Supplementary file 1 — Data S1: Supporting information. [file CCH-52-e70250-s001.pdf]

Group 1: Interview Guide for **Leaders** (e.g., Diagnostic Hub Leads, Directors, Managers, Supervisors)

**Question**
1. What does your diagnostic process look like?<br>o Where are your referrals coming from?<br>o Who are your referrals coming from?<br>o Who else is involved?<br><br>2. What happens internally after a referral is received? (e.g., workflow, scheduling)<br><br>3. How is your team doing with the current process?<br>o What resources does your team have available to them to support autism assessment?<br>o What is going well with the current process (e.g., collaboration, communication)?<br>o What factors are impacting the current process?<br><br>4. Have there been any changes to your approach to autism diagnostic assessment in recent years?<br><br>5. What does the client population look like that you service?<br>o Have there been demographic shifts in your community?<br><br>6. How have these affected pressures for diagnostic assessment?

Group 2. Interview Guide for **Diagnosticians** (e.g., physicians, psychologists)

COM-B	TDF	Question
**Capability**<br><br>Subconstructs:<br><br>psychological or physical	Knowledge	What is your role?<br>Do you know how to diagnose autism?
	Skills (physical, cognitive, inter-personal)	What skills/confidence do you have in diagnosing autism?
	Memory, attention, decision processes	What does your decision-making look like when you meet a child displaying characteristics of autism?
	Behaviour regulation (habits)	How often are you diagnosing autism yourself vs. referring to a specialist? What makes you choose one or the other?
**Motivation**<br><br>Subconstructs:<br><br>automatic, reflective	Social/professional role and identity	To what extent do you think your role contributes to diagnosing autism?
	Beliefs about capabilities	Are you capable of diagnosing autism?
	Beliefs about consequences	How do parents receive your diagnosis of autism?
	Reinforcing behaviour	How does diagnosing autism help families?
	Emotions	How does diagnosing autism make you feel?
	Optimism	Do you feel optimistic that diagnosing autism is within your scope of practice?<br><br>Do you feel adequately supported in diagnosing autism?<br><br>Is autism diagnosis helpful to families in your region?
	Goals	Are you considering any changes to do more diagnosing in your practice?

	Intentions	Do you intend to diagnose autism in your practice?
**Opportunity**<br><br>Subconstructs:<br><br>social or physical	Social Influences	How does your team collaborate when a child requires further assessment?
	Environmental context/resources	What factors are impacting your role in your current process in diagnosing autism? (e.g., workload, education, caseload, continuity of care)<br><br>What is working well in your current process?<br><br>Are there any additional barriers or challenges you experience in your current process?<br><br>What is an ideal state if you could change anything or keep anything the same?

Group 3: Interview Guide for **Clinicians and Community Providers** (e.g., Behaviour Analysts, Family Preservation Workers, Infant Development Workers, Occupational Therapists, Speech-Language Pathologists, Social Workers)

COM-B	TDF	Question
**Capability**<br><br>Subconstructs:<br><br>psychological or physical	Knowledge	What is your role?<br>What knowledge do you have about autism?
	Skills (physical, cognitive, inter-personal)	What skills/confidence do you have in recognizing characteristics of autism?
	Memory, attention, decision processes	What did your decision-making look like when you are concerned with the development of a child in your care? (i.e., referrals)
	Behaviour regulation (habits)	What do you do when you identify a child with features of autism? Do you ever do anything differently? Why?
**Motivation**<br><br>Subconstructs:<br><br>automatic, reflective	Social/professional role and identity	Do you think your role is within the scope of practice to support autism identification?
	Beliefs about capabilities	What do you think will happen if you identify a child with suspected autism?
	Beliefs about consequences	How do parents receive information about your identification of autism characteristics?
	Reinforcing behaviour	What incentives are there for you to identify children as potentially autistic? What disincentives are there for you to identify these children?
	Emotions	How does identifying children with developmental differences or possible autism make you feel??
	Optimism	Do you feel optimistic that identifying children with developmental differences or possible autism can be within your role?<br><br>Do you feel adequately supported in identifying them?

		Is autism diagnosis helpful to families in your region?<br><br>How does a diagnosis autism help families?
	Goals	Are you considering any changes in your work to support autism identification?
	Intentions	Do you intend to support autism identification in your practice/work?
**Opportunity**<br><br>Subconstructs:<br><br>social or physical	Social Influences	How would your same-disciplined colleagues feel about you helping to identify autism? How would your other colleagues feel?<br><br>How does your team collaborate when a child requires screening or further assessment?
	Environmental context/resources	What factors are impacting your role or the current process in supporting autism identification and diagnosis? (e.g., education, workload, caseload)<br><br>What is working well in your current process?<br><br>Are there any additional barriers or challenges you experience in your current process?<br><br>What is an ideal state if you could change anything or keep anything the same?

Group 4. Interview Guide for **Families**

PRE-DIAGNOSTIC ASSESSMENT

1. Before your child was diagnosed, what was your knowledge of how to get an assessment/a diagnosis?

2. Why did you seek help when you did?
   - Did you have a referral, or did you pursue a diagnosis on your own?
   - What did that look like?
   - Did you have a choice in the referral process? (i.e., did family consent to referral?)

3. What options were made available to you to have an assessment and how did you choose, if you had the choice? (i.e., access to funds to pay)

4. What were your feelings about the process in pursuing an assessment/a diagnosis?

DURING DIAGNOSTIC ASSESSMENT (e.g., information gathering via interview, time spent with child):

1. What did the assessment look like? (e.g., the discipline, the assessments used)
   - What did they do well?
   - What do you wish they did differently?
   - What parts of the diagnostic assessment did you understand? What parts did you not understand?

2. How did you feel during the assessment?
   - What did your involvement look like during the assessment? (e.g., interviews, sitting in/sitting out)

3. How did you feel during the delivery of the diagnosis?
   - What did that look like?
   - Who was involved?
   - Was there enough time?
   - Did you understand the outcome?

4. What information were you given regarding next steps? (i.e., services that would be helpful for their child)
   - How confident were you in knowing what to do next?
   - What would have helped increase your confidence?

5. Was the diagnosis helpful in registering for the OAP? Was there any more information required?

FUTURE HOPES:

1. If you were going through this process again, what would you have hoped had been done differently?

2. If you were to design an autism diagnostic pathway/process, what would it look like?

3. What change would you like to see in the process for accessing autism diagnosis moving forward?
